# Serum-derived exosomes promote CD8+ T cells to overexpress PD-1, affecting the prognosis of hypopharyngeal carcinoma

**DOI:** 10.1186/s12935-021-02294-z

**Published:** 2021-10-30

**Authors:** Qian Gao, Hui-Ting Liu, Yu-Qin Xu, Lin Zhang, Yuan-Ru Liu, Qianqian Ren, Ju-ping Sheng, Zhen-Xin Zhang

**Affiliations:** 1 Otorhinolaryngology Head and Neck Surgery Department, Affiliated Hospital of Nantong University, Nantong, China; 2 Medical College of Nantong University, Nantong, China; 3 Traditional Chinese Medicine Hospital of Kunshan, Kunshan, China; 4 Otorhinolaryngology Head and Neck Surgery Department, Haimen District People’s Hospital, Nantong, China

**Keywords:** HNSCC, Immunotherapy, Exosomes, PD-1, Prognosis

## Abstract

**Background:**

Hypopharyngeal cancer (HPC) is associated with a poor prognosis and a high recurrence rate. Immune escape is one of the reasons for the poor prognosis of malignant tumors. Programmed cell death ligand 1 (PD-L1) and programmed cell death-1 (PD-1) have been shown to play important roles in immune escape. However, the role of PD-1/PD-L1 in HPC remains unclear. In this experiment, we investigated the effect of exosomes from HPC patient serum on CD8+ T cell function and PD-1/PD-L1 expression and, thus, on prognosis. We hope to provide guidance for the identification of new targets for HPC immunotherapy.

**Methods:**

PD-1 and CD8 expression in 71 HPC tissues and 16 paracarcinoma tissues was detected by immunohistochemistry. Concurrently, the clinicopathological data of the patients were obtained to conduct correlation analysis. Exosomes were isolated from serum and then identified by Western blotting (WB), transmission electron microscopy (TEM), and nanoparticle tracking analysis (NTA). Flow cytometry was used to assess the activity of CD8+ T cells after exosome stimulation. The effects of exosomes on the ability of CD8+ T cells to kill FaDu cells were assessed by CCK-8 assay. The expression of IL-10 and TGF-β1 was measured by enzyme-linked immunosorbent assay (ELISA). PD-L1 expression in HPC tissue samples was evaluated by immunohistochemistry, and the relationship between PD-1/PD-L1 expression and prognosis was investigated with patient specimens.

**Results:**

PD-1 expression was significantly upregulated on CD8+ T cells in tumor tissues compared with those in normal tissues. The overall survival (OS) and disease-free survival (DFS) of PD-1-overexpressing patients were decreased. Serum exosomes from patients can elevate PD-1 expression on CD8+ T cells and suppress their killing capacity and secretory function. The rate of positive PD-L1 expression was increased in HPC tissues compared with paracancerous tissues. The DFS and OS of the PD-1(+)-PD-L1(+) group were significantly lower than those of the PD-1(−)-PD-L1(−) group.

**Conclusion:**

Our findings indicate that serum exosomes from HPC patients can inhibit CD8+ T cell function and that the PD-1-PD-L1 pathway plays an important role in the immune escape of HPC. Exosomes combined with immunotherapy may guide the treatment of patients with advanced disease in the future.

## Introduction

Head and neck squamous cell carcinoma (HNSCC) encompasses a set of heterogeneous tumors that originate in the epithelium of the oral cavity, oropharynx, larynx and hypopharynx [1]. HPC, which accounts for 3–5% of all HNSCC cases [2], is characterized by rapid growth and metastatic spread, making its treatment difficult [3, 4]. Hence, HPC is considered the most malignant form of HNSCC [5]. Despite advances in therapeutic strategies that combine surgery and chemoradiotherapy, survival has improved only marginally over the past 10 years [6]. Therefore, the search for new therapeutic targets for HPC has broad application prospects in clinical practice.

Recent studies have shown that antitumor immune responses influence the effectiveness of radiotherapy and chemotherapy [7, 8]. After conventional cancer therapy, cytotoxic tumor-infiltrating lymphocytes (TILs) are of great significance for cancer recurrence and metastasis [9, 10]. However, many immunosuppressive mechanisms can inactivate TILs [11, 12]. This suggests that targets that can render TILs dysfunctional will be important for identifying patients with refractory HPC.

PD-1 is an immunosuppressive molecule expressed by lymphocytes [13] and an important marker of T lymphocyte dysfunction [14]. In humans, PD-1 expression by CD8+ T cells in the peripheral blood of patients is altered after viral infection [15]. Recently, the association of elevated PD-1 expression with TIL dysfunction was reported in melanoma, hepatocellular carcinoma, colorectal cancer, and Hodgkin lymphoma [16–19]. These data suggest a phenomenon of tumor-specific T cell unresponsiveness to tumors, which may explain the poor prognosis of some cancers. Thus, we examined PD-1 expression in HPC specimens to investigate its correlation with clinical features.

As carriers of biological information, exosomes are the latest research hotspot and hold great potential in detecting and treating diseases [20, 21]; exosomes are small, nanometer-sized membrane-bound vesicles that can be produced and released by tumor cells and alter the tumor microenvironment by modulating immunity, angiogenesis, and metastasis [22]. It has been demonstrated that IFN-γ stimulation can increase the amount of PD-L1 expressed by these vesicles, which can inhibit CD8+ T cell function and promote tumor growth [23]. This indicated that exosomes may be critical for mediating the immune escape of cancer cells.

In this study, we explored PD-1 expression on CD8+ T cells in HPC tissues and discovered that PD-1 overexpression was a risk factor for patients with HPC by Cox regression analysis. To explore the cause of elevated PD-1 expression, we focused on the effect of serum-derived exosomes on CD8+ T cell function. Subsequently, we found that the PD-1-PD-L1 pathway plays an important role in the immune escape of HPC via recognition of PD-L1 expressed by tumor tissues. These findings provide new insights into immune checkpoint therapy for HNSCC as well as HNSCC biomarker development.

## Materials and methods

### Tissue specimens

Specimens were collected from 71 HNSCC patients who received primary treatment at Nantong University Hospital between August 2009 and April 2020 with complete pathological and clinical means, and 16 normal tissue specimens adjacent to cancer were also collected during the same period. Table 1 summarizes the detailed clinical and medical information of the 71 HNSCC patients. This study was approved by the ethics committee of the Affiliated Hospital of Nantong University.

**Table 1:** The relationship between PD-1 expression and malignancy

Parameter	PD-1 expression rate of CD8+ TILs		
	Low PD-1 CD8^+^ (%) (< 17.5) N = 46	High PD-1 CD8^+^ (%) (> 17.5) N = 25	*P*-value
Age (years)			
< 60	15 (32)	9 (36)	0.592
≥ 60	31 (68)	14 (64)	
Gender			
Male	44 (95)	24 (96)	0.944
Female	2 (5)	1 (4)	
Differentiation			
High differentiation	18 (39)	7 (28)	0.348
Poor differentiation	28 (61)	18 (72)	
Tumor size(cm)			
≤ 2	16 (34)	6 (24)	0.348
> 2	30 (66)	19 (76)	
Clinical stage			
I–II	21 (45)	4 (16)	0.01*
III–IV	25 (55)	21 (84)	
Lymph node metastasis			0.007**
Negative	30 (65)	8 (32)	
Positive	16 (35)	17 (68)	

DFS: disease-free survival; OS: overall survival

*P* values in bold denote significance at < 0.05 level

### Immunofluorescence staining

Paraffin sections of tumor and normal tissues were placed on silanized slides and deparaffinized. After antigen retrieval and the blocking of endogenous peroxidases, the sections were blocked with a working solution of normal sheep serum and incubated with primary antibodies (rabbit anti-PD-1 IgG, Cell Signaling Technology, 861613, 1:100; mouse anti-CD8 IgG, Santa Cruz, sc-1181, 1:100) for 1 h. After three washes with PBS, the slides were then coincubated with secondary antibodies for 1 h. As a final step, the slides were examined under a fluorescence microscope by a pathologist who was blinded to the patients' clinical information. PD-1-positive cells are shown in green (488-conjugated anti-rabbit IgG, Jackson ImmunoResearch, 209-545-088), and CD8+ T cells are shown in red (Cy3-conjugated anti-mouse IgG, Jackson ImmunoResearch, 115-167-003). To analyze the rate of PD-1 expression by CD8+ T cells, we selected and combined random double staining results, and 800 cells from each sample were manually counted and multiplied by the corresponding gray value to quantify the PD-1(+) score of the CD8+ T cells.

### Cell culture

The human HSCC cell line FaDu was purchased from ATCC. FaDu cells were cultured in DMEM with 10% fetal bovine serum and in an incubator at 37 °C with 5% CO_2_.

### Isolation of T-cell subsets

Blood samples were collected from healthy adult volunteers who provided informed consent. Peripheral blood mononuclear cells (PBMCs) were isolated by Ficoll density gradient centrifugation. CD8+ T cells were isolated with a CD8+ T Cell Isolation Kit (Stem Cell, Germany, 100-0185). The purity of the isolated cells was greater than 95%.

### Preparation of exosomes

Serum was extracted from healthy people and patients with HPC, diluted 1:1 with sterile PBS, loaded into 1.5-ml EP tubes, and sequentially centrifuged at 3000*g*, 6000*g*, and 10,000*g* for 30 min each. Then, the supernatants were harvested. The extracted supernatants were filtered through 0.22-µm filters, followed by centrifugation at 140,000*g* for 1 h in an ultracentrifuge, resuspension with 4 ml PBS, and centrifugation again at 140,000*g* for 1 h. Finally, the total protein concentration was quantified by Bradford assay (Sangon Biotech, China, C503041).

### Cellular exosome uptake experiments

To test CD8+ T cell exosome uptake, exosomes were collected from serum and labeled with PKH67. CD8+ T cells and exosomes were incubated for 3 h. After incubation, CD8+ T cells were fixed in 4% paraformaldehyde for 40 min at room temperature. After Hoechst staining of the nuclei, the cellular uptake of the exosomes was observed by confocal laser scanning microscopy (Leica Microsystems, Wetzlar, Germany).

### Western blotting analysis

Tissues and cells were lysed using RIPA buffer. The protein concentrations were determined by BCA assay. 20 µg of proteins were loaded into each well and separated on 10% SDS-PAGE gels before electroblotting onto PVDF membranes. The antibodies used were anti-CD63 (Abcam, ab134045), anti-CD9 (Abcam, ab236630), and anti-GAPDH (Abcam, ab8245) at a dilution of 1:1000. After washing with TBST, the membranes were incubated with secondary antibodies for 1 h. Finally, the bands were visualized by electrochemiluminescence (ECL) and analyzed by Image Software. This experiment was repeated three times.

### Flow cytometric analysis

CD8+ T cells were cultured in 24-well plates at a concentration of 20,000 cells per well. Exosomes from healthy people or patients with HPC were added to the negative control group and the experimental group at a concentration of 100 ng/ml. The cells and exosomes were cocultured for 48 h, and three PBS washes were performed before resuspending the cells in 96 µl PBS. Then, 2 µl of PD-1 and 2 µl of CD8 flow cytometry antibodies were added and incubated on ice at 4 °C for 30 min. Flow cytometry (Becton, Dickinson and Company, America) was used to analyze the cellular immunophenotype, and the data were analyzed.

### ELISA

According to the instructions, TNF-β and IL-10 expression was determined by ELISA kits (FCMACS Nanjing). A standard curve was generated with the absorbance OD value as the ordinate and the concentration of the corresponding TGF-β or IL-10 protein as the abscissa. The data were averaged from three independent experiments.

### Cell killing assay

A total of 5000 FaDu cells were added to each well of a 96-well plate and incubated for 24 h to allow the cells to attach. Then, 2000 T cells stimulated with different exosomes were added and cocultured. The FaDu group, FaDu+ T cell group, FaDu+ exosome-T cell group, and FaDu+ exo-T cell + anti-PD-1 group were established. The PD-1 inhibitor concentration was 4 mg/ml. At 0, 6, 12, 24, and 48 h, 10 µl CCK-8 solution was added to each well, and the absorbance at 450 nm was determined. The specific killing was then calculated as follows: Specific killing % = 100 × [FaDu (OD450) − Experimental Group (OD450)]/[FaDu (OD450)-Black (OD450)].

### Statistical analysis

Student’s t-test was used to evaluate the data. Cancer patients were divided into high PD-1+ -CD8 (> expression score: 17.5%) and low PD-1+ -CD8 (< expression score: 17.5%) groups based on the rates of positive PD-1 expression in the intratumoral T cells. Kaplan–Meier analysis was used to estimate the OS and DFS; univariate and multivariate analyses were performed using the Cox proportional hazards model. All the data analyses were performed using SPSS software version 22.0 (IBM Corp., Armonk, NY, USA). P < 0.05 was considered to be a significant.

## Results

### PD-1 is overexpressed on CD8+ T cells in HPC tissues

**Fig. 1:**
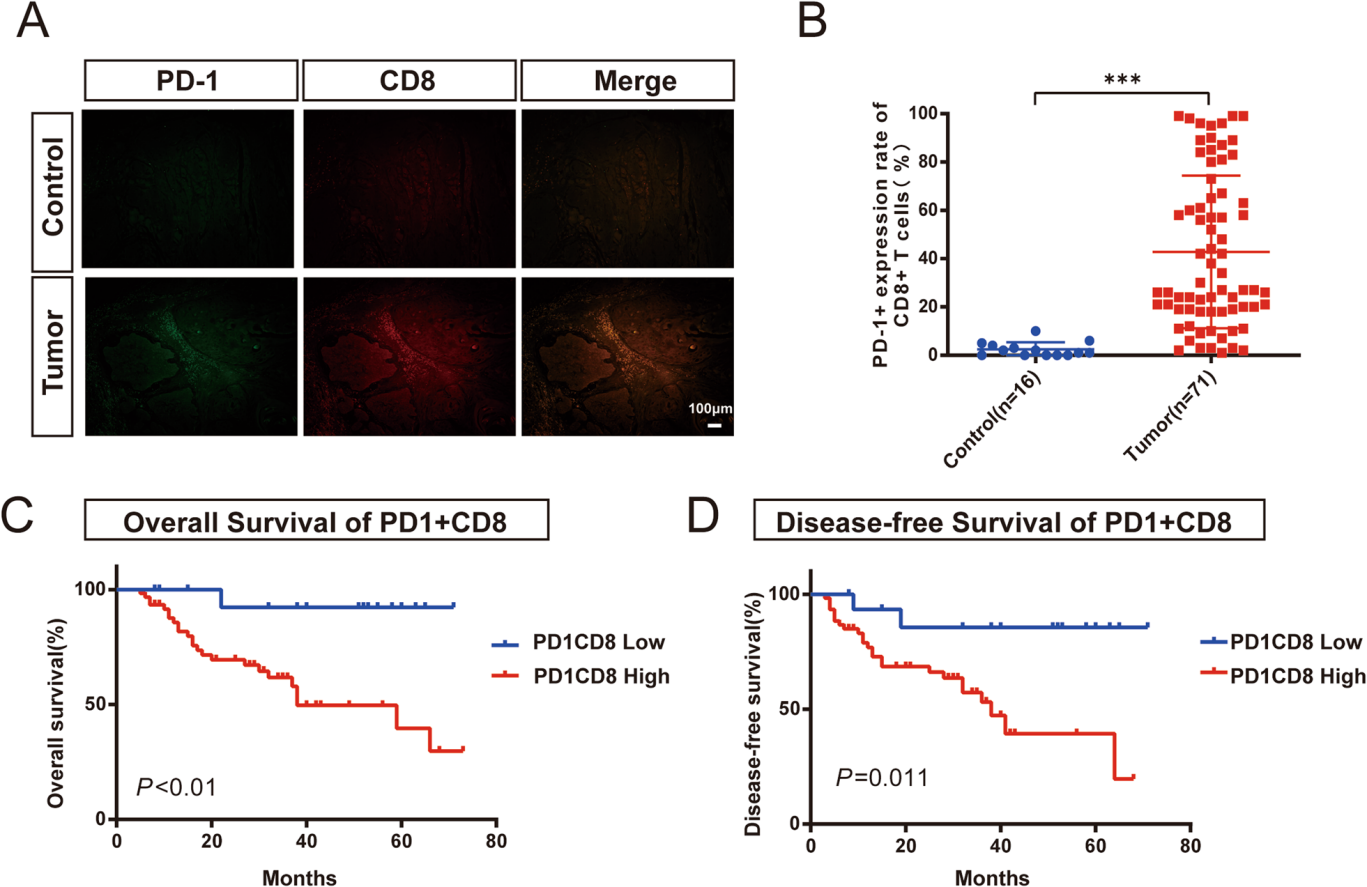
High PD-1 expression in tissues of patients with HPC predicts a worse prognosis. **A** PD-1 is indicated by green fluorescence, and CD8 is indicated by red fluorescence. Scale: 80 µm. Shown at 100× original magnification. **B** Expression of PD-1 in tumor and paracancerous tissues (*P* < 0.001). **C**, **D** Kaplan–Meier survival curves were used to analyze the relationship between PD-1 expression and OS and DFS (*P* < 0.001, *P* = 0.0011)

T cells, which differentiate from lymphoid stem cells in the thymus, are important for human defense against disease [24]. CD8+ cells are an important factor in controlling tumor growth. We detected PD-1 and CD8 expression in paraffin sections using immunofluorescence costaining. PD-1 expression was significantly detected in CD8+ cells in tumor tissue (Fig. 1A), and the average value of PD-1 expression by CD8+ T cells was higher (Fig. 1B).

### High PD-1 expression by intratumoral CD8+ cells predicts poor outcome of HPC

First, we analyzed the possible relationship between PD-1 expression and malignancy (Table 1). According to the rate of positive PD-1 expression in CD8+ T cells, patients were divided into two subgroups: a high expression group (> 17.5% positive rate, n = 25) and a low expression group (< 17.5% positive rate, n = 46). The results showed no statistically significant correlations between PD-1 expression and sex, age or differentiation. However, PD-1 expression was closely correlated with lymph node metastasis and clinical stage (*P* < 0.005). Overall, higher PD-1 expression in CD8+ T cells predicted earlier lymphatic metastasis and worse clinical grade of patients.

To verify the relationship between PD-1 expression and prognosis, we conducted a follow-up of the patients. The median follow-up time of all patients was 34 ± 19 months. Univariate analysis showed that significant predictors of OS were PD-1+ CD8 T cells (*P* = 0.027) and lymph node metastasis (*P* = 0.031), and significant predictors of DFS were PD-1+ CD8+ T cells (*P* = 0.023) and lymph node metastasis (*P* = 0.033) (Table 2). Moreover, higher PD-1 expression on CD8+ T cells was significantly associated with worse OS and DFS (Fig. 1C, D), which was consistent with the multivariate analysis results (Table 3). Overall, these results indicated that PD-1 may be an independent risk factor for HPC.

**Table 2:** Univariate analysis to assess the association of clinicopathological parameters with prognosis of HPC

Parameter	OS		DFS	
	Hazard ratio (95% CI)	*P*-value (Cox)	Hazard ratio (95% CI)	*P*-value (Cox)
PD-1 CD8^+^ T cell	9.664 (1.299 ~ 71.899)	0.027*	5.447 (1.269 ~ 23.371)	0.023*
Sex	22.567 (0.016 ~ 32,759)	0.401	1.847 (0.248 ~ 13.672)	0.551
Age	2.410 (0.943 ~ 6.161)	0.066	1.141 (0.511 ~ 2.549)	0.748
Differentiation	2.342 (0.930 ~ 5.897)	0.071	2.899 (1.163 ~ 7.222)	0.022*
Tumor size	1.139 (0.502 ~ 2.586)	0.755	1.306 (0.585 ~ 2.916)	0.515
Clinical stage	5.203 (1.221 ~ 22.168)	0.026*	3.973 (1.189 ~ 13.273)	0.025*
Lymph node metastasis	4.492 (1.534 ~ 13.155)	0.031*	2.559 (1.077 ~ 6.077)	0.033*

DFS: disease-free survival; OS: overall survival

*P* values in bold denote significance at < 0.05 level

**Table 3:** Multivariate analysis to assess the association of clinicopathological parameters with prognosis of HPC

Parameter	OS		DFS	
	Hazard ratio (95% CI)	*P*-value	Hazard ratio (95% CI)	*P*-value
PD-1+ CD8+ T cell	5.575 (0.704 ~ 44.145)	0.104	3.068 (0.606 ~ 15.538)	0.176
Differentiation	–	–	1.559 (0.368 ~ 6.604)	0.546
Clinical stage	1.737 (0.366 ~ 8.248)	0.487	1.528 (0.585 ~ 3.993)	0.387
Lymph node metastasis	2.880 (0.938 ~ 8.844)	0.065	2.130 (0.836 ~ 5.428)	0.113

DFS: disease-free survival; OS: overall survival

*P* values in bold denote significance at < 0.05 level

### CD8+ T cells took up serum-derived exosomes

**Fig. 2:**
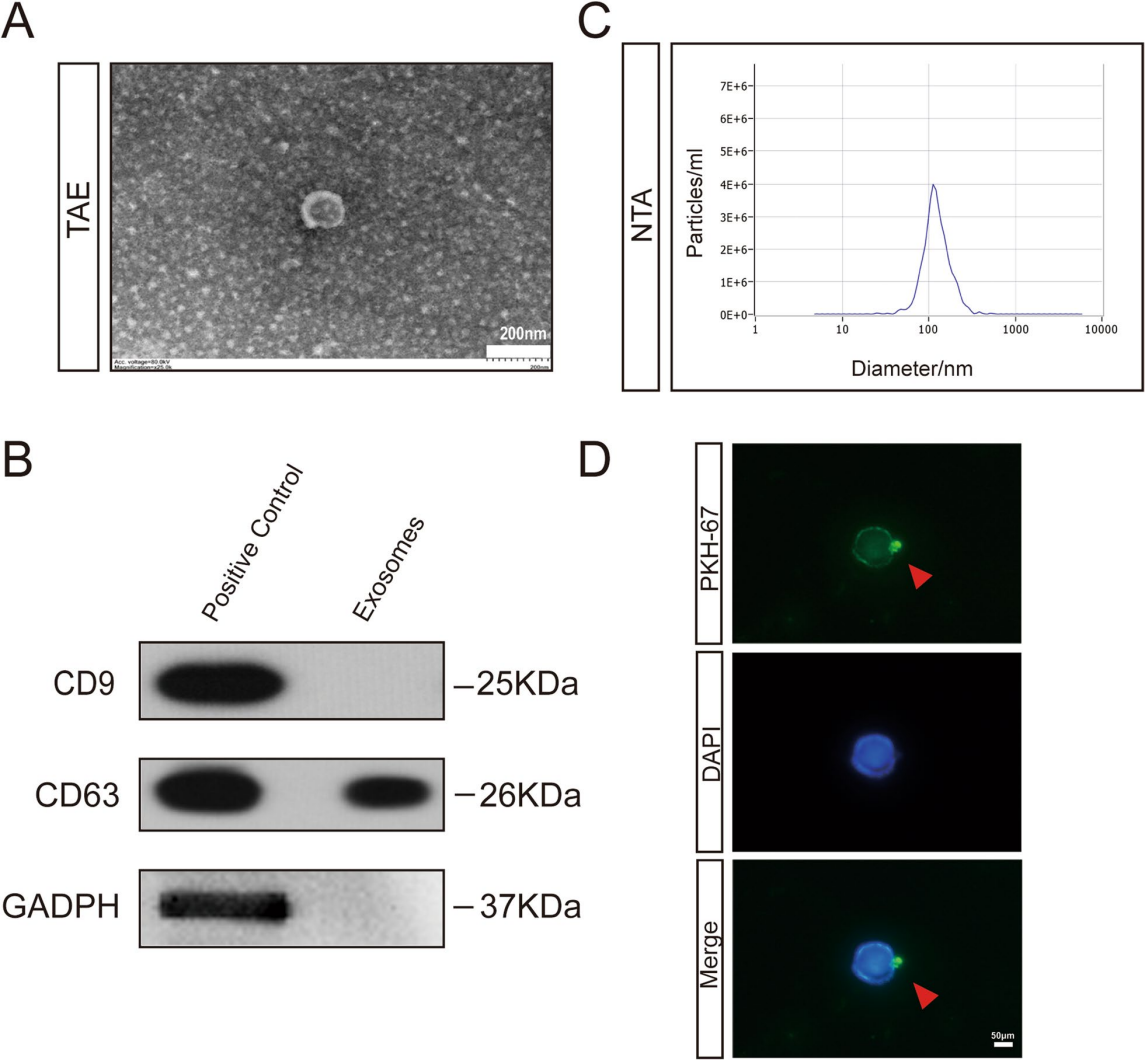
Serum-derived exosomes can be taken up by CD8+ T cells. **A** Representative TEM images of purified exosomes from sera of patients with HPC. Scale: 200 nm. **B** Immunoblotting of CD63 expression in whole cell lysates and purified exosomes. **C** The exosomes were identified by TEM. **D** The exosomes could be taken up by CD8+ T cells. The exosomes were stained with PKH-69 (green), and the nuclei were stained with DAPI (blue). The red arrow indicates the exosomes. Scale: 50 nm

It has been reported that exosomes play a major role in immune regulation [25]. To explore the influence of exosomes on CD8+ T cells, we extracted exosomes from the serum of HPC patients. TAE showed that the exosome vesicles had diameters ranging from 40 to 100 nm (Fig. 2A). CD9 and CD63 are surface markers of exosomes [26], while GAPDH is a cell-specific structural protein. We confirmed the identity of the exosomes according to the presence of these markers (Fig. 2B). Similarly, NTA revealed a mean exosome size of 115.9 nm (Fig. 2C). To confirm that serum exosomes could be transferred into CD8+ T cells, we cocultured CD8+ T cells and exosomes. Our study found that exosomes were taken up by CD8+ T cells and were mainly distributed in the cytoplasm (Fig. 2D). This result suggested that exosomes can be used as carriers of signals between HPC cells and immune cells.

### Serum-derived exosomes from patients with HPC can affect CD8+ T cell function

**Fig. 3:**
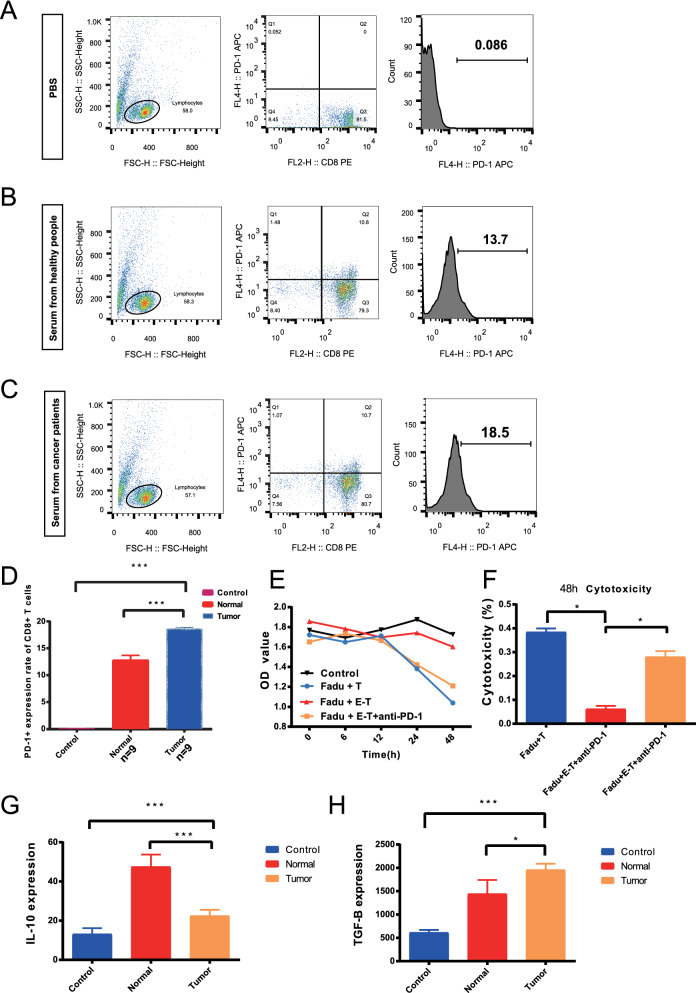
Serum-derived exosomes can affect the function of CD8+ T cells. **A**–**C** Changes in PD-1 expression in CD8+ T cells stimulated with PBS, control serum exosomes and patient serum exosomes were observed. **D** The serum-derived exosomes of patients significantly increased the expression of PD-1 in CD8+ T cells (*P* < 0.001). **E**, **F** The cell killing ability of T cells was determined by CCK-8 assay (*P* = 0.049). **G**, **H** The levels of IL-10 and TGF-β in the medium were determined by ELISA (*P* < 0.001, *P* < 0.001; *P* < 0.001, *P* = 0.393)

We used exosomes (100 ng/ml) isolated from the sera of different patients to stimulate CD8+ T cells (1*10^5) for 48 h and found that serum exosomes from patients with advanced cancer could significantly increase PD-1 expression by CD8+ T cells (Fig. 3A–D). Moreover, CD8+ T cells treated with exosomes were cocultured with hypopharyngeal carcinoma cells, and the cell killing ability of CD8+ T cells was detected by CCK-8 assay within 48 h. Our study revealed that patient serum-derived exosomes significantly reduced the killing capacity of CD8+ T cells against FaDu cells (Fig. 3E). In addition, the killing ability of CD8+ T cells could be obviously restored by the addition of a PD-1 inhibitor (Fig. 3F).

IL-10 is the most common proinflammatory factor and is also a representative inflammatory factor secreted by T cells, which is of interest for T cell secretory function [27]. Furthermore, TGF-β is an inhibitory molecule on immune cells in the microenvironment [28]. We next assessed the amount of IFN-β in the cocultured supernatants by ELISA. The results revealed a significant increase in IL-10 secretion in the cancer serum-derived exosome-treated group but no significant change in TGF-β secretion (Fig. 3G, H).

### PD-1-PD-L1 may be an important pathway affecting prognosis

**Fig. 4:**
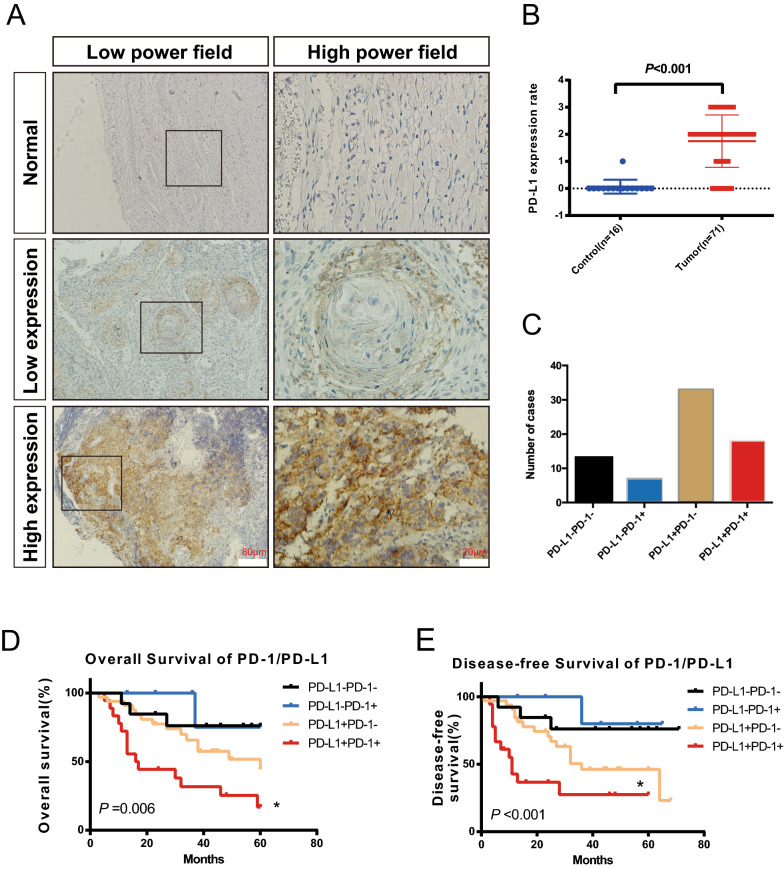
Expression of PD-L1 in tissues of patients with hypopharyngeal carcinoma. **A** Typical images of no, low and high PD-L1 expression in patient and normal tissues. **B** The expression of PD-L1 in tumor tissues was significantly higher than that in normal tissues (*P* = 0.005). **C** Statistics of patients grouping. **D**, **E** Kaplan–Meier survival curves were used to analyze the relationship between PD-1/PD-L1 expression and OS and DFS

PD-1-PD-L1 is a classical T cell inhibition pathway [29]. In this study, we observed PD-L1 expression in 16 paracancerous tissues and 71 cancer tissues. Histochemical analysis revealed that PD-L1 expression in HPC was significantly higher than that in normal tissues (*P* < 0.001) (Fig. 4A, B). In addition, we divided the patients into the PD-1 (−)/PD-L1 (−) group, PD-1 (−)/PD-L1 (+) group, PD-1 (+)/PD-L1 (−) group and PD-1 (+)/PD-L1 (+) group (Fig. 4C) and analyzed the prognosis of each group. Significantly, the survival rate of the PD-1 (+)/PD-L1 (+) group was much lower than that of the PD1 (−)/PD-L1 (−) group in terms of the OS rate and DFS rate. The results indicated that PD-L1 and PD-1 have a certain synergistic effect, which has an important effect on the survival rate of patients (Fig. 4D, E). Overall, it is reasonable to suspect that the PD-1/PD-L1 pathway plays an important role in the progression of HPC and is significantly correlated with prognosis.

## Discussion

**Fig. 5:**
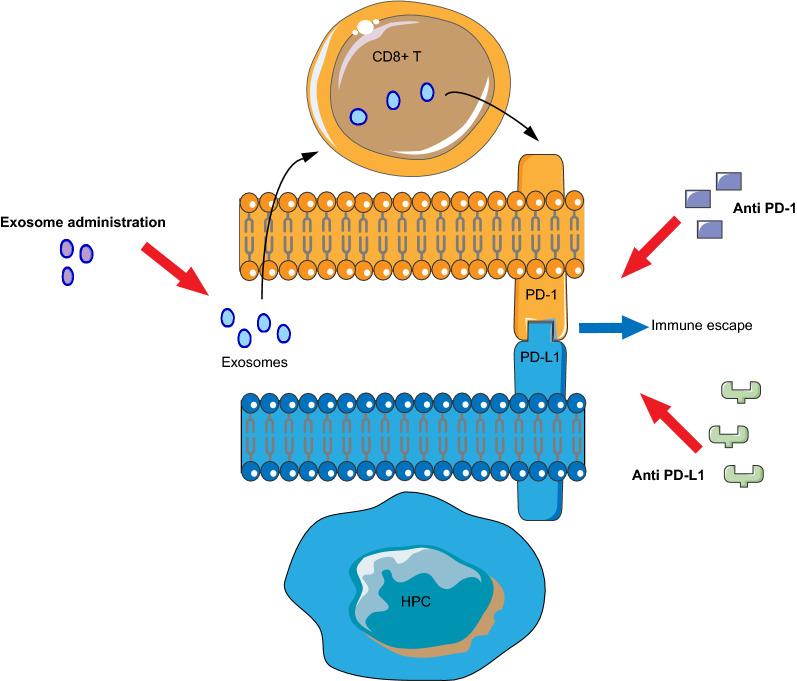
The serum-derived exosomes of patients protect HPC cells from CD8+ T cells through up-regulation of PD-1

In this study, the results demonstrated that PD-1 expression by CD8+ T cells in HPC tissues was higher than that in adjacent tissues, and high PD-1 expression in CD8+ T cells can be used as an independent risk factor for predicting adverse clinical outcomes of mortality, treatment failure and local recurrence. Furthermore, we also showed that serum-derived exosomes can increase PD-1 expression in CD8+ T cells (Fig. 5), and PD-1/PD-L1 pathway activation is important for HPC immune escape.

T cells are a major component of the immune response and can induce a persistent immune response, especially against infectious diseases and tumors [30]. T cell exhaustion is one of the hallmarks of cancer, and typical characteristics of exhausted T cells are as follows: increased inhibitory receptor expression, such as PD-1 and CTLA4, which leads to a low response to cancer cells [31]. Upregulation of PD-1 expression is a natural consequence of T cell activation and is required for the termination of immune responses.

To investigate the mechanism underlying the regulatory phenotype in CD8+ T cells, we explored exosomes derived from the serum of multiple patients. Exosomes are cellular trafficking systems that are formed by the release of intracellular endosomes via exocytosis [22]. Cancer cells often use this transport system to regulate and dominate immune cell processes, as well as communication with other cancer cells. Studies have shown that exosomes secreted by tumor cells can induce T cell apoptosis and promote immune escape [32]. Moreover, impairment of the antitumor immune response prevents the clearance of residual cancer cells, thereby increasing the risk of recurrence.

Previous studies have reported that multiple immunomodulatory mechanisms limit tumor cell suppression and clearance in HPC [33, 34]. Since most of these mechanisms operate through the suppression of effector T cells, we hypothesized that markers of immunosuppression would help identify patients with high risk HPC. PD-1 is a candidate marker because it is not only a specific receptor for T cell inhibition but also a target of T cell dysfunction [35]. The results of the present study revealed that CD8+ T cells are selectively exhausted under the influence of tumor exosomes, which may explain the correlation between PD-1 and cancer recurrence. Additionally, the weak association between PD-1 expression and distant metastasis implies that the immunosuppressive state of the primary tumor site is unrelated to the state of the metastatic site. However, we did not study tumor tissue from metastatic sites, which might be the direction of our further research.

The balance between PD-1 and PD-L1 determines the final fate of T cell activation or peripheral tolerance [36]. PD-1 and PD-L1 expression has been described in patients with cancers of the liver [37], bladder [38], colon [39], cervix [40], ovary [41], and breast [42], and the expression of this molecule can serve as a reliable predictor for gastric, esophageal, and breast cancer [43]. Currently, PD-1/PD-L1 pathway inhibitors have been tested for efficacy in a variety of tumor types, including ovarian, breast, bladder, colorectal, follicular lymphoma, gastric cancer and diffuse large B cell lymphoma [37–43]. Anti-PD therapy has become central to immunotherapy of human tumors, especially solid tumors. Therefore, PD-1/PD-L1 blockade therapy may become a new comprehensive treatment for patients with moderately advanced HPC (Fig. 5).

Functional experiments of exosomes on CD8+ T cells showed that local tumor immunosuppression was reversible. Therefore, the strong immunosuppression of HPC can be alleviated by targeting PD-1 and PD-L1, which improves not only the efficacy of tumor immunotherapy but also that of traditional HPC therapy.

In conclusion, our study elucidated the basic functions and highlighted the mechanisms of exosomes and PD-1 in the immunotherapy of HPC, which will hopefully guide the future clinical diagnosis and development of combination therapies for HPC.

## Data Availability

The data that support the findings of our research are available from Affiliated Hospital of Nantong University, but restrictions apply to the availability of these data, which were used under license for the current study, and so are not publicly available.

## Abbreviations

HPC: Hypopharyngeal cancer
PD-L1: Programmed cell death ligand 1
PD-1: Programmed cell death-1
PD-2: Programmed cell death-2
TEM: Transmission electron microscopy
NTA: Nanoparticle tracking analysis
WB: Western blot
ELISA: Enzyme-linked immunosorbent assay
OS: Overall survival
DFS: Disease‑free survival
HNSCC: Head and neck squamous cell carcinoma
TILs: Tumor-infiltrating lymphocytes
PBMCs: Peripheral blood mononuclear cells
BCA: Bicinchoninic acid
ECL: Electrochemiluminescence
CTLA4: Cytotoxic T lymphocyte associated protein 4
